# The Contribution of Numerical Magnitude Comparison and Phonological Processing to Individual Differences in Fourth Graders’ Multiplication Fact Ability

**DOI:** 10.1371/journal.pone.0158335

**Published:** 2016-06-30

**Authors:** Tamara M. J. Schleepen, Hanneke I. Van Mier, Bert De Smedt

**Affiliations:** 1 Department of Cognitive Neuroscience, Faculty of Psychology and Neuroscience, Maastricht University, Maastricht, The Netherlands; 2 Faculty of Psychology and Educational sciences, Parenting and Special Education Research Unit, KU Leuven, Leuven, Belgium; Ghent University, BELGIUM

## Abstract

Although numerical magnitude processing has been related to individual differences in arithmetic, its role in children’s multiplication performance remains largely unknown. On the other hand, studies have indicated that phonological awareness is an important correlate of individual differences in children’s multiplication performance, but the involvement of phonological memory, another important phonological processing skill, has not been studied in much detail. Furthermore, knowledge about the relative contribution of above mentioned processes to the specific arithmetic operation of multiplication in children is lacking. The present study therefore investigated for the first time the unique contributions of numerical magnitude comparison and phonological processing in explaining individual differences in 63 fourth graders’ multiplication fact ability (mean age = 9.6 years, *SD* = .67). The results showed that children’s multiplication fact competency correlated significantly with symbolic and nonsymbolic magnitude comparison as well as with phonological short-term memory. A hierarchical regression analysis revealed that, after controlling for intellectual ability and general reaction time, both symbolic and nonsymbolic magnitude comparison and phonological short-term memory accounted for unique variance in multiplication fact performance. The ability to compare symbolic magnitudes was found to contribute the most, indicating that the access to numerical magnitudes by means of Arabic digits is a key factor in explaining individual differences in children’s multiplication fact ability.

## Introduction

Arithmetic abilities such as adding or subtracting numbers are crucial for successful participation in educational and daily life settings. The basis for arithmetic skills is laid in childhood and marked individual differences in mathematical competence are already apparent in this period of life [1]. Recently, there has been a growing interest in the cognitive factors that underlie such individual differences. On the one hand, the ability to process numerical magnitudes has been found to be an important domain-specific factor in the development of mathematics, for a review see [2, 3]. On the other hand, cognitive abilities such as working memory, e.g. [4, 5], phonological processing, e.g. [6, 7], and processing speed, e.g. [8, 9], have been identified as crucial domain-general factors.

Although most studies have focused on broad measures of mathematical competence, an increasing number of studies has addressed more specific arithmetical skills, such as single-digit addition and subtraction, e.g. [10–12]. Surprisingly only a few studies have focused on the origins of individual differences in multiplication. For example, De Smedt, Taylor, Archibald and Ansari [13] investigated the role of phonological processes in 9–11 year-olds’ multiplication performance, revealing unique associations between phonological awareness and multiplication. To the best of our knowledge, no study has specifically investigated the role of numerical magnitude processing in multiplication. Against this background, the present study investigated for the first time the association between numerical magnitude processing and multiplication. In this way, we aimed to extend prior research investigating relations between numerical magnitude processing and addition and subtraction. Another aim of the study was to examine whether numerical magnitude processing explains unique variance in multiplication over and above the variance explained by phonological processing.

Please note that in our study multiplication problems are used that are created by single-digit numbers. We will use the term multiplication fact(s) when we are referring to literature and results related to the use of such multiplication problems. When we are referring to the operation of multiplication in general we will use the term multiplication. For reasons of brevity, when we describe, analyse and discuss the arithmetic task that was used, we will refer to the task simply as the multiplication task.

### Multiplication

Multiplication is a central arithmetic skill in elementary school curricula that in most Western countries is introduced in the second grade (i.e. 7/8 years) and that is extensively practiced up till grade four (i.e. 9/10 years). Already in the early stages of multiplication learning, children are encouraged to memorize the multiplication tables. As such, the association between a problem and its corresponding answer is stored in long-term memory [14]. Already by the end of the second grade, the majority (i.e. 60–90%) of single-digit multiplication problems is solved by direct memory retrieval [15]. It has been suggested that multiplication facts are most likely represented in long-term memory as phonological codes, e.g. [16], which are formed when memory associations between problem-answer pairs are strengthened during arithmetic practice. With increasing age and through schooling, these problem-answer pair representations in long-term memory become stronger. Approximately from sixth grade onwards, children have established a memory network similar to that of adults, including the (basic) multiplication tables [14].

### The role of numerical magnitude comparison in multiplication

Many studies have examined the role of numerical magnitude processes in explaining individual differences in children’s arithmetic abilities, for review see [2, 3] and more specifically in arithmetic fact retrieval [12], although this work is mainly restricted to addition and subtraction. In the study of Vanbinst and colleagues [12], it was reported that third-grade children with better access to numerical magnitudes via Arabic digits (which was measured by means of a symbolic magnitude comparison task), retrieved more facts from memory and were more fluent in using fact retrieval and procedural strategies during single-digit addition and subtraction. Yet, no study so far has investigated the association between numerical magnitude processes and children’s performance of multiplication facts, which is a prototypic example of fact retrieval since it is the major strategy on these problems.

The ability to process numerical magnitudes is typically measured with dot and digit magnitude comparison tasks that require participants to decide which of two numerosities is the largest. Nonsymbolic (dot) magnitude comparison skills are thought to reflect the acuity of the approximate number system (ANS). This is a language-independent system that is present from young infancy and shared across species, enabling the estimation of quantities [17]. Symbolic (digit) magnitude comparison skills are believed to index an exact symbolic representation system, which is language-dependent, develops gradually over the school years and allows for processing of discrete numbers [18].

A central question is whether the representation of numerical magnitudes (e.g. dots) itself or the access to it via Arabic digits is more important for arithmetic. To distinguish between these two alternatives, performance on a nonsymbolic and a symbolic magnitude comparison task is typically evaluated. If the nonsymbolic as well as the symbolic task are related to arithmetic skills, this provides evidence for the view that numerical magnitude processing itself is most crucial for arithmetic performance. If arithmetic ability is only related to symbolic magnitude comparison, this supports the idea that the access to numerical magnitude via Arabic digits is the strongest predictor of arithmetic. Evidence has been presented for both views [12, 19–28], but in a recent meta-analysis it was shown that in particular the access to numerical magnitude representations via Arabic digits is important for arithmetic [3].

Importantly, the above mentioned studies that investigated the importance of symbolic and nonsymbolic processing often used general arithmetic achievement tests. These tests only yield an average score, reflecting performance across several different arithmetic operations (e.g. addition, subtraction, multiplication and division). Therefore these studies do not reveal any information about the relation between children’s symbolic/nonsymbolic magnitude comparison and multiplication in particular. Thus, although many studies have examined the importance of numerical magnitude processing in arithmetic and more recently in addition/subtraction, its role in the performance of multiplication facts remains unknown. Whereas addition and subtraction problems are solved either by direct fact retrieval or by using procedural strategies (e.g. counting) [29], it has been suggested that direct fact retrieval is the main strategy used in solving multiplication problems, e.g. [30]. For example, Imbo and Vandierendonck [15], showed that in fourth graders the percentage of fact retrieval use was about 80% in multiplication, whereas it was 60% in addition. The current study thus goes beyond previous ones by investigating for the first time the role of nonsymbolic and symbolic numerical magnitude comparison in children’s multiplication fact competency, an arithmetical operation typical of using fact retrieval strategies.

Furthermore, until now, relatively little is known about the determinants of retrieving multiplication facts. As said, it is generally assumed that problems consisting of multiplication facts are solved by a process of rote memorization, but it remains to be determined if, and to what extent, numerical representations play a role herein. The present study aimed to shed light on this question. We expect that magnitude comparison plays a role in arithmetic (i.e. multiplication) because of the way multiplication facts are stored in long-term memory. It is generally agreed that arithmetic facts are stored in a semantic form in an interrelated network of arithmetic facts [31]. An important feature of this network is that arithmetic facts are meaningfully organized in long-term memory [32]. Against this background, it could be that “magnitude” is an important candidate according to which meaning is assigned to stored multiplication facts. Hence, it might well be that individuals with better magnitude comparison skills, thus children who are faster in deciding which of two presented magnitudes is the largest, can possibly more easily access number-semantic arithmetic fact representations in long-term memory.

### The role of phonological processing in multiplication

Behavorial as well as neuroimaging studies have demonstrated that phonology plays an important role in multiplication, e.g. [33], most likely because the multiplication tables are stored in memory as phonological (verbal) codes [16]. Yet, until now there is only one study that specifically addressed to what extent phonological processes explain individual differences in children’s multiplication fact competency [13]. In this study the role of two phonological abilities was investigated, namely phonological awareness, referring to an individual’s sensitivity and access to the sound structure of language, and phonological memory, involving the short-term storage of phonological speech sounds [34]. De Smedt and coworkers [13] demonstrated that phonological awareness was uniquely related to 9–11 year-olds fact retrieval abilities (i.e. in solving multiplication and small addition/subtraction problems), independent of individual differences in phonological short-term memory or intellectual ability. However, in this study only phonological awareness was directly correlated to multiplication performance, while phonological memory was correlated with an averaged arithmetic score (including performance on addition, subtraction and multiplication problems). Therefore it still remains to be investigated to which extent phonological processes play a role in children’s ability to retrieve multiplication facts and if and which of these processes, phonological awareness and/or phonological memory is most related to multiplication facts.

Also, in the study of De Smedt et al. [13] the role of phonological memory was measured with a repetition task consisting of non-words. The lack of a significant correlation between this task and the arithmetic processes measured in this study (addition, subtraction and multiplication) might be due to the fact that this task only measures memory for (non)words. Because studies have reported that numerical measures of memory are frequently related to arithmetic [35], we also included in our study a task that measures memory for numerical information.

### The present study

The current study examined the role of numerical magnitude comparison in children’s multiplication fact ability. We investigated both the role of nonsymbolic and symbolic magnitude comparison. In this way the current study will contribute to the debate on whether the numerical representation itself or the access to it by means of Arabic digits is the most important factor contributing to multiplication fact performance. Additionally, the association between phonological processes (i.e. phonological awareness and phonological memory) and children’s multiplication fact ability was examined to investigate if numerical magnitude comparison accounts for unique variance over and above phonology, and vice versa. To address the above questions, fourth-grade children performed a multiplication task, a symbolic and nonsymbolic magnitude comparison task and several phonological awareness and (short-term/working) memory tasks. To rule out that any possible relations between the processes of interest would in fact be due to individual differences in general reaction time and/or intellectual ability, all children also performed a motor reaction time task and the Raven’s standard progressive matrices test (reflecting intellectual ability) as control measures.

## Methods

### Participants

Participants were 63 fourth-grade children (31 boys) with a mean age of 9.6 years (*SD* = .67, age range 9–11 years) for whom written parental consent was obtained prior to testing. This age group was selected because children in fourth-grade have mastered all (single-digit) multiplication tables. All 63 children that had parental permission to participate in the study were included. None of them had a history of learning difficulties or diagnosed developmental disorders. Children were recruited from four different primary schools that were located in suburban areas in the South of the Netherlands. The majority of children were Caucasian and from middle class families. The study was approved by the ethical committee of the Faculty of Psychology and Neuroscience of Maastricht University and was performed in accordance with the ethical standards as laid down in the 1964 Declaration of Helsinki.

### Tasks

The multiplication task, the numerical magnitude comparison tasks, and the motor reaction time task were computerized and programmed and presented via the software package Presentation (Neurobehavioral Systems, Albany, CA; www.neurobs.com). A 15-inch laptop was used to administer these tasks and children were seated at a viewing distance of approximately 70 cm from the laptop screen. All visual stimuli were presented in white (Font size 60) against a black background. In each of these tasks, two keys of the keyboard were used as response keys: the “D” for the left side and the “L” for the right side. The response keys were labelled with white stickers and children were instructed to keep their index fingers on both keys during task administration. Children performed five practice trials for each computerized task to familiarize them with the task assignment. Before a task was started, children received an explanation of the task and were instructed and encouraged to respond as quickly and accurately as possible. In the computerized tasks, accuracy as well as reaction time was measured.

### Multiplication task

This task comprised all single-digit multiplication tables from 2–9 with the exception of tie problems (e.g. 3 × 3) and problems containing 0 or 1 as operand. This resulted in 56 multiplication problems, which were administered in 2 blocks of 28 trials each. An equal number of small and large problems were presented. Small problems involved problems in which the products of the operand were smaller or equal to 25 whereas large problems were defined as problems in which the products of the operand was larger than 25. The beginning of a trial was cued with a 250 ms rectangle, followed by a blank screen for1000 ms. A multiplication problem in Arabic format was then presented horizontally for 2000 ms after which the multiplication problem was replaced by an equal sign that appeared for 500 ms, followed by two response alternatives displayed on the right and left side of the screen, one correct and one incorrect. Children were instructed to press the response key corresponding to the side where the correct answer was shown. The response alternatives remained on screen until a response was given. Incorrect answers were table related and were created by adding or subtracting 1 multiplicand to or from the correct answer. The position of both the correct answer and largest operand was balanced across problems.

### Numerical magnitude comparison tasks

#### Nonsymbolic magnitude comparison

In this task, children had to indicate the larger of two simultaneously presented dot arrays, displayed at the left and right side of the screen, by pressing the response key on the side of the numerically larger dot array. All possible combinations of the numerosities 1–9 were presented, resulting in a total of 72 trials (which were administered in two blocks of 36 trials each). The dot arrays were generated with the MATLAB script provided by Piazza, Izard, Pinel, Le Bihan and Dehaene [36] and were controlled for non-numerical parameters, such as dot size, total occupied area, and density. On one half of the trials, dot size, array size, and density were positively correlated with number, and on the other half of the trials, dot size, array size, and density were negatively correlated. While in the former the more numerous array had larger dots and occupied a larger area, in the latter the more numerous array had smaller dots and occupied a smaller area. This was done to prevent that participants would mainly base their decisions on non-numerical cues or perceptual features. A trial started with a 250 ms white rectangle presented in the centre of the screen. The dot arrays appeared 1000 ms later and to prevent counting of the dots, they disappeared after 860 ms.

#### Symbolic magnitude comparison

In this task, children had to select the largest of two simultaneously shown Arabic digits, appearing on the left and right side of the screen, by pressing the key on the side where the numerically larger one appeared. The stimuli were all combinations of the numerosities 1–9, resulting in 72 trials. Children performed two blocks of 36 trials each. A white rectangle (250 ms) displayed in the center of the screen cued the presentation of the Arabic digits, which were presented after 1000 ms and disappeared when the child responded.

### Phonological processing tasks

#### Phonological awareness

The Phoneme Analysis Test (FAT), consisting of the Phoneme Deletion and the Phoneme Exchange subtest, was used as a measure of phonological awareness [37]. In the Phoneme Deletion test, children were asked to repeat an existing word but had to say what the word would be if the first letter of the word would be deleted. For example, children heard the word *tiger* and were expected to say *iger*. In the Phoneme Exchange (i.e. spoonerism) task, children were asked to switch the initial letters from two words, e.g. they heard the words *music lesson* and were expected to say *lusic messon*. All items of the FAT were spoken by a native female speaker of the Dutch language and had been prerecorded and were presented auditory via a laptop. Both subtests were preceded by three practice items to familiarize children with the task. Each subtest comprised 12 items. The experimenter pushed a response button on the computer immediately after the child answered, and then indicated if the given answer was correct. The number of correctly solved problems (max = 12 per subtest) was used as the dependent measure.

#### Phonological memory

Digit span forward and backward tasks and the nonword repetition task were included to index phonological memory. The digit span forward and backward tests, adapted from the Wechsler Intelligence scale for children (WISC-III, Dutch version; [38], were used to index phonological short-term memory and working memory for numerical information, respectively [39]. In the digit span forward and backward tasks, children heard a series of digits which they had to recall immediately in the same (i.e. forward digit span test) or reverse (i.e. backward digit span test) order of presentation. The series of digits were read by the experimenter at a rate of 1 per sec. There were two trials for each span length, which ranged from two to nine digits and increased every two trials with one digit. The task was terminated when the child failed to correctly recall two consecutive series of a span length. The number of correctly recalled sequences represented a child’s short-term memory span (digit span forward) or working memory-capacity (digit span backward).

A Dutch nonword repetition (NWR) task, designed by Rispens, Baker and Duinmeijer [40], was used as a purer measure of phonological short-term memory. This test entailed the immediate repetition of nonwords. To minimize reliance on long-term phonological representations, all nonwords consisted of novel phonological forms which had low Dutch word likeness. Children first received an explanation of the task, followed by three practice items. A total of 40 items were presented in two blocks of 20 items each. The task was to repeat each nonword immediately after presentation. The responses of the children were recorded with a voice recorder and scored offline. All items of the nonword repetition task, which were spoken by a native female speaker of the Dutch language, had been prerecorded and were presented auditory via a laptop. For each item, the number of correctly recalled phonemes was calculated, because research has shown that this is a more sensitive scoring measure than counting the number of correctly recalled words [41].

### Control measures

#### Reaction time

A motor reaction time task was included to control for children’s reaction time on the keyboard. In this task, children were shown two simultaneously presented figures, one on the right and one on the left side of the screen. One figure was black and the other was white. Children were instructed to indicate where the white figure was shown by pressing the response key on the side where it appeared. A total of 20 trials were presented. A trial started with a 250 ms white rectangle which was followed 1000 ms later by the black and white figures which remained on the screen until the child responded.

#### Intellectual ability

The Raven’s standard progressive matrices were included to control for intellectual ability [42]. This paper and pencil test consisted of 60 items divided over 5 sets (A, B, C, D and E) with 12 items per set. Each item consisted of a visual pattern from which a piece was missing. Children were instructed to select the missing piece out of 6 (sets A and B) or 8 pieces (sets C, D and E) displayed below the pattern. One point per correctly solved item was assigned. In the current study raw scores were used.

### Procedure

Children were tested in two sessions during regular school hours. In the first, group-based session, that lasted about 30–45 minutes, the Raven test was administered. In the second session, that included individual testing of each child in a quiet room (lasting approximately 45 minutes), the numerical magnitude comparison tasks, the phonological processing tasks, the multiplication task and the motor reaction time task were performed in a fixed order (i.e. the numerical magnitude comparison tasks, the motor reaction time task, the multiplication task, the FAT, the digit span forward and backward and the NWR-task). All tasks were administered by trained experimenters. Between tasks, children were given the opportunity to take a short break. Children were rewarded with a small present upon completion of both sessions.

## Results

In all computerized tasks, only trials for which correct responses were given were included in the reaction time (RT) analyses.

### Descriptive analyses

The means and standard deviations of the administered tasks are displayed in Table 1. In the multiplication task, all children performed above chance level (50%). To test possible differences in reaction times and/or accuracy rates depending on problem-size, paired t-tests were performed comparing small vs. large problems. Both for reaction time and accuracy, the typical problem-size effect was found, showing increased reaction times and decreased accuracy rates for large vs. small multiplication problems (reaction time: *t*(62) = -6.59, *p* < .001; accuracy: *t*(62) = -5.54, *p* < .001, respectively).

**Table 1:** Descriptive statistics for the administered measures.

Measure	*M*	*SD*
Multiplication fact retrieval		
Small problems		
Reaction time (ms)	926.17	458.75
Accuracy (%)	93.25	6.97
Large problems		
Reaction time (ms)	1308.68	762.46
Accuracy (%)	87.19	10.75
Numerical magnitude comparison		
Nonsymbolic		
Reaction time (ms)	705.83	252.54
Accuracy (%)	94.49	5.94
Symbolic		
Reaction time (ms)	659.05	127.03
Accuracy (%)	95.68	3.01
Phonological processing		
Phonological awareness		
FAT Phoneme deletion (maximum score: 12)	10.94	1.19
FAT Phoneme exchange (maximum score: 12)	10.75	1.22
Phonological memory		
Digit span forward (maximum score: 16)	9.22	2.04
Digit span backward (maximum score: 14)	4.92	1.59
Nonword repetition (maximum score: 321)	275.57	16.04
Control measures		
Motor reaction time task (ms)	471.23	84.15
Raven (maximum score: 60)	37.73	7.39

*Note*. All scores represent raw scores

To verify the presence of *distance effects* in the magnitude comparison tasks, we calculated for each child the regression slope for which reaction time was predicted by numerical distance. As expected and in line with prior research [43], the slope of the symbolic (i.e. mean slope = -22.7ms, *SD* = 17.9) and nonsymbolic (mean slope = -27.6ms, *SD* = 29.4) task was negative, meaning that reaction time and distance were negatively related to each other. Furthermore, both the slopes of the symbolic and nonsymbolic task differed significantly from zero, *t* (62) = -10.1, *p* < 001, and, *t* (62) = -7.4, *p* < 001, respectively.

*Size effects* were also present in the symbolic and nonsymbolic magnitude comparison task, as shown by larger reaction times when comparing large (e.g. 7 vs. 9) vs. small numbers (e.g. 1 vs. 2), *t* (62) = -6.7, *p* < .001, and, *t* (62) = -5.9, *p* < .001, respectively.

As accuracy levels were at ceiling level in the multiplication task and the numerical magnitude comparison tasks, only the response times (solely of correctly answered trials) on these tasks were included in subsequent correlational and hierarchical regression analyses.

Accuracy measures on the phoneme deletion and phoneme exchange tests of the FAT were very similar, with an accuracy of 10.9 for the former and 10.7 for the latter subtest. Furthermore, a positive correlation was found between the accuracy of the phoneme deletion task and the accuracy of the phoneme exchange task (*r* = .43, *p* < .001) and similar results were obtained when separately computing correlations for each of the two phonological awareness tasks and the speed of solving multiplication problems. We therefore averaged over both tasks and the averaged FAT score was included in subsequent analyses.

### Correlational analyses

Pearson correlation coefficients were computed between all administered tasks (see Table 2). To control for multiple comparisons, we applied the False Discovery Rate (FDR) correction using the Benjamini-Hochberg procedure to all correlational analyses [44].

**Table 2:** Pearson correlations between the administered measures (N = 63).

		1	2	3	4	5	6	7	8
1.	Reaction time multiplication								
2.	Reaction time nonsymbolic task	.54**							
3.	Reaction time symbolic task	.61**	.60**						
4.	Accuracy FAT	-.19	-.14	-.20					
5.	Digit span forward	.14	.07	.07	.29*^(1)^				
6.	Digit span backward	.09	-.04	-.04	.36**	.35**			
7.	Nonword repetition	-.29*^(1)^	-.13	-.09	.34**	.53**	.24		
8.	General reaction time	.39**	.51**	.64**	-.04	.12	-.09	-.10	
9.	Intellectual ability	-.03	-.08	-.09	.14	.16	.23	.23	-.20

*Note*. The reaction time on the multiplication task was averaged across small and large problems. The accuracy on the phoneme deletion and phoneme exchange test was averaged into one single FAT score.

^(1)^ These correlations were not significant after the FDR-correction for multiple comparisons.

* *p* < .05

** *p* < .01

Because the correlations were very similar for small and large problem sizes, we averaged the performance on both multiplication problems. Further, there was a non-significant (negative) correlation between reaction times and accuracy in the multiplication task (*r* = -.16, *p* = .22), suggesting that there was no speed-accuracy trade-off. For correlations including RT measures, additional partial correlation analyses were performed including reaction time on the motor reaction time task as a covariate. As can be seen in Table 2, significant correlations were found between the reaction time on the nonsymbolic and symbolic magnitude comparison tasks, as well as between the digit span forward and digit span backward tasks, and between the digit span forward and NWR-task performance. Correlations between the multiplication task and numerical magnitude comparison and phonological tasks will be described below.

#### Correlations between numerical magnitude comparison and multiplication

Significant positive correlations were found between the overall reaction time of both the symbolic and nonsymbolic magnitude comparison task and the speed of solving multiplication fact problems. These correlations indicate that children who were faster in processing symbolic and nonsymbolic magnitudes, were also faster in solving multiplication fact problems. These correlations remained significant after controlling for general reaction time (*r*s > .43, *p* < .01, uncorrected p-value) and remained significant after FDR-correction.

#### Correlations between phonological processing and multiplication

No significant correlation was found between phonological awareness, as measured by accuracy on the FAT, and performance of multiplication facts. Separate correlations computed between the reaction time for multiplication facts and accuracy on the two subtests of the FAT (i.e. phoneme deletion and phoneme exchange), were also not significant (phoneme deletion-multiplication: *r* = -.18, *p* = .15; phoneme exchange-multiplication: *r* = -.14, *p* = .28, uncorrected p-values).

As for the correlation between phonological memory and retrieving multiplication facts the following results were obtained. There were no significant correlations between the reaction time in the multiplication task and the digit span forward or backward scores. A negative significant correlation was found between the nonword repetition (NWR) score (reflecting the total number of correctly recalled phonemes) and the speed of solving multiplication problems. This correlation indicates that children who correctly recalled more phonemes from nonwords, were faster in solving multiplication problems. However, this association was not significant anymore after application of the FDR correction.

### Hierarchical regression analysis

The above mentioned (uncorrected) correlational analyses showed that both numerical magnitude comparison and phonological processing (i.e. phonological short-term memory, NWR-task performance) were related to multiplication in fourth graders. To examine the amount of unique variance accounted for by each variable in children’s multiplication fact performance and to examine if numerical magnitude processing explained significant variance in retrieving multiplication facts beyond the variance explained by phonology, while controlling for possible variance explained by intellectual ability and general reaction time, a hierarchical regression analysis was performed. The results of the hierarchical regression analysis for multiplication facts are shown in Table 3.

**Table 3:** Hierarchical regression analysis testing if numerical magnitude processing explained significant variance in retrieving multiplication facts over and above phonological processing (N = 63).

*Step*	*Variable(s)*	*Β*	*t*	Δ*R*²
1	Intellectual ability	-.03	-.20	.00
2	General reaction time	.41	3.35**	.16
3	Nonword repetition task	-.28	-2.38*	.07
4	Reaction time symbolic task	.48	3.49**	
	Reaction time nonsymbolic task	.25	2.01*	.24

*Note*. The values for the predictors entered in step 1 and step 2 represent the values without the predictors entered in step 3 and step 4

* *p* < .05

** *p* < .01

In the hierarchical regression analysis, in step 1, the Raven’s scores were included to control for differences in intellectual ability. In the 2^nd^ step we included the response times on the motor reaction time task to control for differences in general reaction time. In the 3^rd^ step, scores on the nonword repetition task (indexing phonological processing) were included. In step 4 scores on the symbolic and nonsymbolic magnitude comparison task were included, which allowed us to examine if numerical magnitude processing explained significant variance in retrieving multiplication facts beyond the variance explained by phonological processing. This analysis revealed that introducing numerical magnitude comparison in step 4 explained an additional 24.4% of the variance in multiplication performance, and this change in *R*^2^ was significant, *F*(2,57) = 13.3, *p* < .001. This indicates that numerical magnitude comparison indeed explains additional variance over and above phonology while also holding constant the influence of processing speed and intellectual ability.

**Fig 1:**
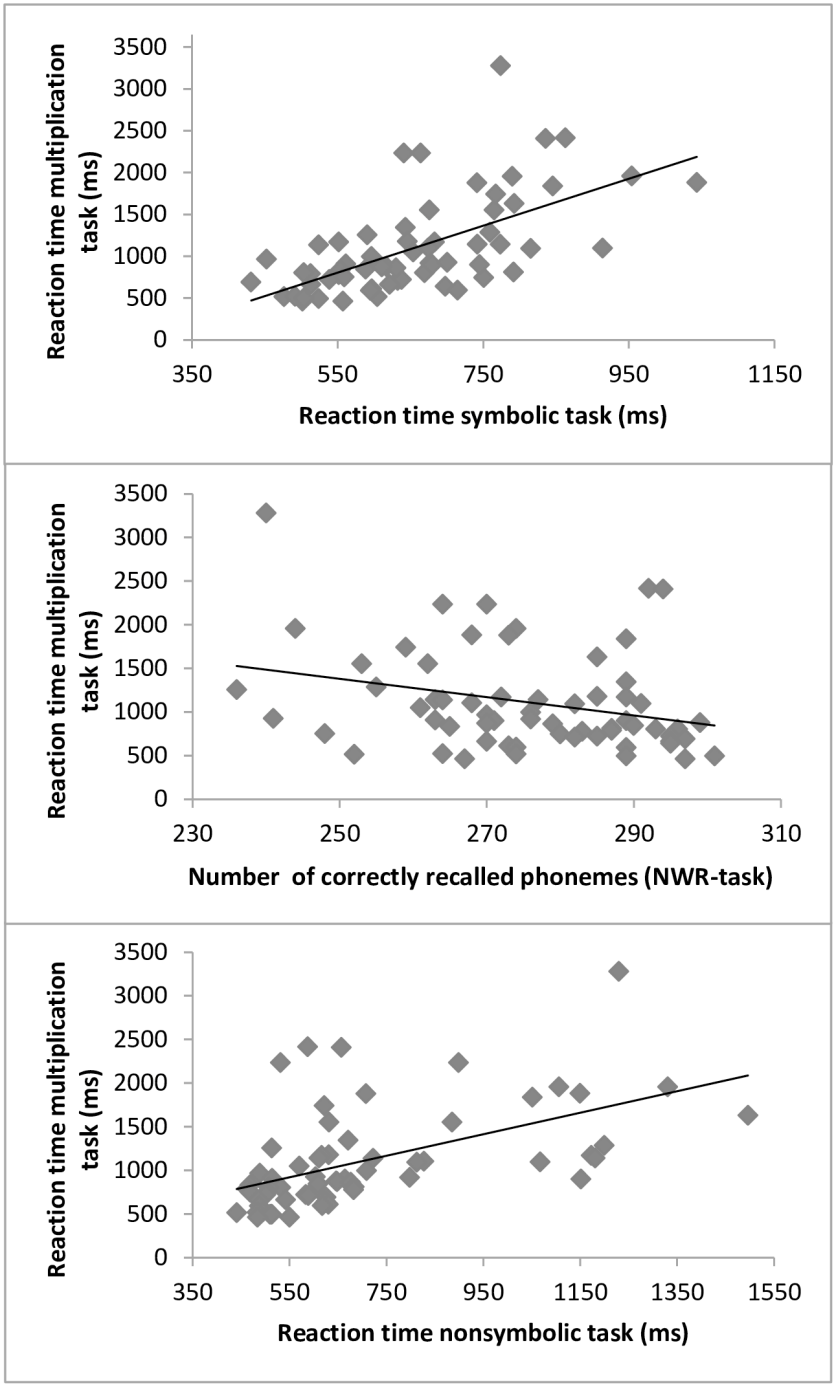
Scatterplots showing the association between children’s reaction time on the multiplication task with a) the reaction time on the symbolic task, b) the number of correctly recalled phonemes in the NWR-task, and c) the reaction time on the nonsymbolic task. The solid black line in the scatterplots depicts the linear relationship between the two measures.

Scatterplots showing the relation between the speed of multiplication facts and the above mentioned factors are shown in Fig 1. As can be seen in this figure, there were no major outliers that might have influenced the observed correlations.

Next to the hierarchical regression analysis described above and based on the finding that of both magnitude comparison tasks, symbolic magnitude comparison had the highest beta weight, we performed an additional regression analysis in which we statistically tested if symbolic magnitude comparison contributed significant variance over and above nonsymbolic magnitude comparison and vice versa. This was done by adding symbolic and nonsymbolic magnitude comparison in separate steps in the regression analysis. In a first regression analysis, nonsymbolic magnitude comparison was added in step 4 and symbolic magnitude comparison added in step 5. This analysis showed that symbolic magnitude comparison explained an additional 11.2% of the variance over and above nonsymbolic magnitude comparison, which was statistically significant, *F*(1,57) = 12.2, *p* < .01. In a second regression analysis, symbolic magnitude comparison was added in step 4 and nonsymbolic magnitude comparison was added in step 5. This analysis revealed that also nonsymbolic magnitude comparison explained unique variance (3.7%, which was statistically significant, *F*(1,57) = 4.0, *p* = .049). Note that the change in explained variance was smaller when nonsymbolic comparison was entered in the last step (3.7%) than when symbolic magnitude comparison was added in the last step (11.2%). Thus, this indicates that although both symbolic and nonsymbolic magnitude comparison explained unique variance in retrieving multiplication facts, symbolic magnitude comparison contributed more to individual differences in multiplication performance.

### Additional analyses

It is known that two different numerical systems are used when comparing nonsymbolic quantities, i.e. the object tracking system (OTS) for the fast and accurate enumeration of small number sets (3/4 items) and the approximate number system (ANS), which supports the representations of larger (>4) number sets, which is less accurate and depends on the ratio of the two presented numerical sets [45]. Furthermore, recent studies indicate that cognitive control might be another cognitive process involved in comparing nonsymbolic magnitudes, in particular in incongruent trials in which the number of dots is negatively related to the area they occupy, that explains the association between nonsymbolic number comparison and mathematical performance [46]. We therefore tested if the correlation between multiplication fact performance differed depending on the OTS/ANS mechanism and congruency. Performance in the multiplication task (reaction times) was correlated with the reaction time on four different types of trials in the nonsymbolic task (see Table 4, also for the mean reaction times and standard deviations for each trial type): 1) congruent trials within the subitizing (OTS) range, 2) congruent trials beyond the subitizing (ANS) range, 3) incongruent trials within the subitizing (OTS) range, and 4) incongruent trials beyond the subitizing (ANS) range. This analysis revealed that all four trial types significantly correlated with multiplication fact performance (all *r*s between .51 and .65, all *p*s < .001) showing that all four trial types displayed more or less similar correlations with multiplication fact retrieval. Moreover, we re-analyzed the above described regression analyses that included the nonsymbolic task, where in each of the regression analysis performance on the nonsymbolic task was replaced by each of the four trial types (i.e. in separate analyses due to multicollinearity among the trial types). These analyses showed similar results.

**Table 4:** Mean reaction times (and standard deviations) in the four different trial types in the nonsymbolic comparison task depending on the OTS/ANS mechanism and congruency and their associations with the speed of multiplication facts.

Measure			Multiplication (RT)
	*M*	*SD*	
Congruent–Subitizing RT (ms)	712.39	264.82	.58**
Congruent- Non-subitizing RT (ms)	845.80	346.44	.57**
Incongruent–Subitizing RT (ms)	709.54	228.11	.51**
Incongruent- Non-subitizing RT (ms)	808.24	389.32	.66**

Note.

** *p* < .01

To test whether the correlation between symbolic magnitude comparison ability and multiplication fact performance was mainly driven by trials with a small numerical distance, we computed correlations between the reaction times on trials with a small numerical distance (i.e. 1) and multiplication fact performance on the one hand and between the reaction times on trials with a large numerical distance (i.e. calculated by averaging the reaction times on distances 6,7 and 8) and multiplication fact performance on the other hand. This analysis showed that multiplication fact performance showed a more or less similar correlation with trials including a small numerical distance (*r* = .57, *p* < .001) or a large numerical distance (*r* = .54, *p* < .001). Additionally, also for the symbolic task the above-reported regression analyses were performed again, where symbolic task performance was replaced either by trials with a small or large numerical distance. These analyses yielded the same results as reported above.

## Discussion

Although there is ample evidence that children’s numerical magnitude processing contributes to individual differences in arithmetic abilities [2, 19–27, 47] and recently in addition/subtraction [12], its role in multiplication fact retrieval has not been examined. To fill this gap, the current study examined the association between numerical magnitude processing skills and multiplication fact ability in fourth-grade children. To extend the existing knowledge regarding the role of phonological processing in multiplication facts, correlations between phonological awareness and phonological memory scores and multiplication fact performance were computed. A hierarchical regression analysis was performed to answer the question if numerical magnitude processing contributed to individual differences in multiplication fact retrieval and if it uniquely explained variance over and above the variance explained by phonological processing (awareness), a factor that was found in previous research to be associated with children’s multiplication fact competency [13].

The results add to the existing literature in several ways. Firstly, it was shown that both symbolic and nonsymbolic magnitude processing skills were associated with children’s multiplication fact performance. Secondly, symbolic magnitude processing, phonological short-term memory and nonsymbolic magnitude processing accounted for unique variance in fourth-graders multiplication fact ability, even when individual differences in intellectual ability and general reaction time were controlled for. The ability to process symbolic magnitude explained the most variance, indicating that children’s access to numerical magnitudes via Arabic digits is an important determinant of individual differences in multiplication fact performance.

In previous studies the ability to process symbolic and nonsymbolic magnitudes has been identified as an essential domain-specific factor of general mathematical skills, e.g. [2, 25, 48]. The current results extend these findings by showing that these associations are also observed when specifically investigating multiplication facts. These associations indicate that children who are faster in accessing symbolic and nonsymbolic representations, are also faster in solving multiplication fact problems.

Although multiplication fact performance was significantly related with the symbolic and nonsymbolic task, the results of the hierarchical regression analysis showed that the ability to compare two Arabic digits emerged as the most significant predictor of multiplication fact performance, even after controlling for individual differences in general reaction time and intellectual ability. An earlier study that examined the association between numerical magnitude processing and fact retrieval in 8-year-old children also reported a unique role for symbolic magnitude comparison in solving subtraction and addition problems [12, 49]. The current study adds to these findings, by demonstrating that in particular symbolic magnitude processing is important in fourth-graders multiplication fact performance.

One of the main reasons why especially the ability to compare two Arabic digits is essential to multiplication facts might be that symbolic magnitude comparison and multiplication fact retrieval share the same semantic mapping processes. That is, when deciding which of two presented digits is the largest in symbolic magnitude comparison, participants must have a good understanding of the exact magnitude represented by each Arabic digit. This requires that the symbols are semantically linked to their (nonsymbolic) representations in a precise manner. Likewise, when solving a multiplication fact problem, subjects must retrieve the correct answer from memory. As mentioned before, these problem-answer pairs are most likely stored in memory as verbal (semantic) codes. The hypothesis that symbolic magnitude comparison and retrieval of multiplication facts involve comparable semantic coupling processes is supported by neuroimaging research demonstrating that these skills activate overlapping brain regions, including the left (superior) temporal/parietal region. In adult participants, fMRI measurements showed that this region was involved in processing the semantic connection between a symbolic digit and its corresponding magnitude [50]. Increased practice related activation in the left temporo-parietal cortex has been observed when adult participants learned complex multiplication problems [51]. In an fMRI study in children, Prado, Mutreja and Booth [33] found increased activation in this region during processing the semantic connection between a single-digit multiplication problem and its answer.

The ability to process nonsymbolic magnitudes emerged as another factor explaining unique variance in children’s multiplication fact skills (over and above symbolic magnitude processing), although this factor contributed much less than symbolic magnitude processing. Recently, it has been observed that the association between the nonsymbolic task and arithmetic performance was only found when analysing incongruent trials in the nonsymbolic task in which there was a conflict between the number of stimuli and the area they occupied (i.e. when more dots occupied a smaller area) [46], which suggests that cognitive control accounts for the relation between nonsymbolic task performance and arithmetic. In light of these findings, it may be that also in the current study cognitive control processes (partly) explain the relation between performance in the nonsymbolic task and multiplication fact skills. Indeed, when individuals are presented with a multiplication problem, often multiple candidate answers are activated that require inhibition of the incorrect answers (e.g. when shown 6 x 4 =, answers belonging to the same table, like 5 x 4 = and 7 x 4 = are also activated but need to be inhibited to be able to give the correct answer). The hypothesis that cognitive control played a role in nonsymbolic magnitude comparison was tested in the current study by analysing the different trials in the nonsymbolic comparison task, including incongruent trials. The results showed that the relation between nonsymbolic magnitude comparison and retrieval of multiplication facts was not mainly driven by the incongruent trials. This suggests that the role of cognitive control processes is probably limited, at least in this study. On the other hand, it has to be acknowledged that we did not employ any independent measures to index cognitive control and the analyses of the incongruent trials may not have been too sensitive enough, as our study was not originally designed to investigate congruency effects. Future research is thus needed to shed more light on the exact role of cognitive control in the relation between nonsymbolic task performance and multiplication fact retrieval.

Previous research has shown that different numerical mechanisms are used when comparing nonsymbolic magnitudes (OTS and ANS) [45]. In the current study the outcome of additional analyses showed that the relation between nonsymbolic magnitude comparison and multiplication facts was not dependent on the size of the number. However, due to limited variability in the accuracy data in the current study, the analyses performed to assess the OTS and ANS were restricted to the reaction time data. This is not optimal since usually assessment of the OTS is based on reaction time analysis, while accuracy (error) analysis is typically employed for assessment of ANS acuity. Future research should therefore use numerical magnitude tasks with large numerosities to ensure sufficient variability in the accuracy data to index ANS acuity.

With respect to the debate regarding the importance of symbolic and/or nonsymbolic magnitude comparison in relation to arithmetic, and in particular to multiplication facts, our results are consistent with the idea that both competencies play a role in multiplication fact retrieval in fourth graders, although symbolic magnitude comparison seems to be most important for multiplication fact performance.

Besides the contribution of domain-specific factors in the performance of multiplication facts, also domain-general factors such as phonological processing have been shown to play an important role in arithmetic and more recently in multiplication fact retrieval [13]. Yet, the unique role of phonological processing beyond the influence of numerical magnitude processing remained unclear. The present study aimed to resolve this issue, with the results of the hierarchical regression analysis showing that among the phonological measures included only the NWR-task accounted for unique variance in multiplication facts, over and above the variance explained by the numerical magnitude comparison measures. This means that children with better abilities to shortly store phonological codes were more fluent in solving multiplication problems. Importantly, it should be mentioned that only a small amount of variance (i.e. 0.7%) was explained by performance in the NWR-task. Also, the correlation between the NWR-task and multiplication facts was not significant anymore after the FDR-correction. Note however that the FDR correction was fairly conservative since 36 correlations were included in the FDR-correction (i.e. all measures included in the current study were correlated with each other), while only 8 correlations were directly relevant for our research questions. Thus, on this basis we feel that it is justified to conclude that the NWR-task (measuring phonological memory) does make a significant contribution to multiplication fact retrieval, albeit we acknowledge that this contribution is modest in the current study.

Previous studies have implicated phonological skills in individual differences in children’s fact retrieval and multiplication skills [13, 33, 52], but have not concurrently considered the possible contribution of numerical magnitude processing. The current data extend this research by demonstrating that even when controlling for the influence of numerical magnitude processing, phonological processing still plays a (modest) role in children’s multiplication fact performance. When solving multiplication fact problems, phonological short-term memory might thus be responsible for shortly storing and retrieving phonological information from memory, i.e. the answer to a multiplication fact problem.

The role of phonological processing in multiplication fact retrieval in the current study was smaller than in previous studies in the same age range [13]. This might be explained by differences in language. Indeed, prior studies reporting a link between phonological processing and multiplication were run in English-speaking children, the language of which is not transparent in its letter-sound mappings. The current participants, on the other hand, were Dutch speaking children, whose language is much more transparent. As a result, the NWR-task used in the current study possibly loads to a lesser extent on phonology than NWR tasks used in prior studies with English-speaking children. This may explain the weaker link we found between the NWR task and multiplication facts in our study.

In contrast to De Smedt et al. [13], the current study did not find a relation between phonological awareness and multiplication facts. Similar as above, it could be that the phonological awareness task used in the study of De Smedt et al. [13] placed higher demands on phonological awareness skills than the task used in the current study (and thus had greater predictive power [53], due to differences in transparancy between languages. Additionally, the current measure of phonological awareness ability was derived from an average score of two phonological awareness subtests (i.e. phoneme deletion and phoneme exchange), whereas in De Smedt et al. [13] only the phoneme deletion task was used to index phonological awareness. But note that also when computing correlations separately with the phoneme deletion task, the correlation between multiplication facts and phoneme deletion remained nonsignificant in the current study.

How can we explain that only performance in the NWR-task but not performance in the digit span forward task, another phonological short-term memory measure, was associated with multiplication facts? This may be so because performance in the NWR-task is, in addition to the short-term maintenance of information, dependent on the quality of phonological representations, e.g. [54–59], a factor earlier associated with children’s multiplication skill [13]. Indeed, when performing the NWR-task, having (access to) precise phonemic representations helps subjects to memorize and correctly recall the non-existing words. The digit span forward task probably draws to a lesser extent on existing phonological representations as only (known) digits need to be memorized. This explanation is supported by the correlation analyses (see Table 2), showing a higher correlation between the NWR-task and the FAT than between the digit span forward task and the FAT. However, it could also be that due to a smaller variance in the digit span forward task compared to the NWR-task, multiplication fact retrieval correlated only with the NWR-task, but not with the digit span forward task.

The current study did not show a relation between digit span backward performance (i.e. indexing working memory) and multiplication facts. This might be due to the fact that the children included in this study (fourth graders) had already largely automatized (i.e. had memory representations of) the multiplication tables, so that demands on working memory were only minimal. This is in line with studies showing that already at the age of 7–8 years, children solve more than 60% of the multiplication problems by directly retrieving the answer from memory [15, 30, 60]. In accord with the above, Simmons and colleagues [61] demonstrated that also in 7–8 year-olds, phonological working memory was not associated with multiplication performance.

Finally, the current study’s findings might have important implications for children with developmental dyscalculia, who have specific problems with retrieving (multiplication) facts [62, 63]. On a speculative basis, one implication of the present study’s findings is that poor numerical magnitude comparison skills are a possible cause for the problems children with dyscalculia experience in retrieving multiplication facts. This would suggest that training (symbolic) magnitude comparison skills might lead to better multiplication fact retrieval abilities in dyscalculics. However, training studies are needed in order to test this claim.

### Conclusion

The present study is the first to examine the role and relative, unique contribution of numerical magnitude comparison skills and phonological skills in fourth-graders multiplication fact ability. Individual differences in multiplication fact performance were significantly associated with measures of symbolic and nonsymbolic magnitude comparison, and phonological short-term memory. The ability to process symbolic magnitudes explained the most variance in fourth graders multiplication fact ability, followed by phonological short-term memory and nonsymbolic magnitude processing. It remains to be investigated if symbolic magnitude comparison and phonological memory are predictively related to children’s multiplication fact ability (i.e. if having good symbolic magnitude comparison and phonological memory skills is a precursor for developing good multiplication skills); this should be the focus of future longitudinal research. In future studies, it would be interesting to examine the effects of interventions on children’s multiplication fact skills, aimed at improving (symbolic) magnitude comparison and/or phonological memory skills.
